# High volume online hemodiafiltration: a global perspective and the Brazilian experience

**DOI:** 10.1590/2175-8239-JBN-2023-0104en

**Published:** 2023-12-22

**Authors:** Maria Eugenia Fernandes Canziani, Jorge Paulo Strogoff-de-Matos, Murilo Guedes, Ana Beatriz Lesqueves Barra, Sinaia Canhada, Luciana Carvalho, Douglas Gemente, Carlos Eduardo Poli-de-Figueiredo, Roberto Pecoits-Filho

**Affiliations:** 1 Universidade Federal de São Paulo, São Paulo, SP, Brazil.; 2 Universidade Federal Fluminense, Faculdade de Medicina, Niteroi, RJ, Brazil.; 3 Pontifícia Universidade Católica do Paraná, Curitiba, PR, Brazil.; 4 Fresenius Medical Care, Rio de Janeiro, RJ, Brazil.; 5 B Braun, São Paulo, SP, Brazil.; 6 Pontifícia Universidade Católica do Rio Grande do Sul, Departamento de Medicina, Porto Alegre, RS, Brazil.

**Keywords:** Hemodiafiltration, Dialysis, Renal Insufficiency, Chronic, Kidney Failure, Chronic., Hemodiafiltração, Diálise, Insuficiência Renal Crônica, Falência Renal Crônica

## Abstract

Online hemodiafiltration (HDF) is a rapidly growing dialysis modality worldwide. In Brazil, the number of patients with private health insurance undergoing HDF has exceeded the number of patients on peritoneal dialysis. The achievement of a high convection volume was associated with better clinical imprand patient – reported outcomes confirming the benefits of HDF. The HDFit trial provided relevant practical information on the implementation of online HDF in dialysis centers in Brazil. This article aims to disseminate technical information to improve the quality and safety of this new dialysis modality.

## RESUMO

A hemodiafiltração (HDF) on-line é uma modalidade dialítica em rápido crescimento no mundo. No Brasil, o número de pacientes com planos de saúde privados tratados por HDF já ultrapassa aquele de pacientes em diálise peritoneal. O alcance de um alto volume convectivo associado à redução de desfechos clínicos e do risco de morte confirmam os benefícios da HDF. Dados nacionais do estudo HDFit forneceram informações práticas relevantes sobre a implementação da HDF on-line em clínicas de diálise no Brasil. O objetivo desta publicação é a disseminação de informações técnicas que possam auxiliar na utilização, com qualidade e segurança, dessa nova modalidade dialítica.

## Introduction

Hemodiafiltration (HDF) is a dialysis mode that combines convection and diffusion to remove uremic toxins of different molecular weights^1,2^. HDF has been known for more than four decades, and its use has grown significantly, especially in European countries, Australia and Japan^3,4^. Several randomized clinical and observational trials have demonstrated the safety and efficacy of HDF in removing uremic toxins and decreasing the occurrence of cardiovascular events^5–8^. Despite this, there is still a wide debate in the literature about the benefits of HDF when compared to high flux hemodialysis (HD). Recently, the CONVINCE randomized clinical trial, in which patients on high convection volume HDF (23L/session) were compared against subjects on high-flow HD, reported a 23% reduction in the risk of death from all causes (primary endpoint) in patients on high convection volume HDF^9^. The publication of secondary endpoints and sub-analyses of this study and the conclusion of the H4RT (High-volume HDF versus High-flux HD Registry Trial)^10^, scheduled for 2025, should provide enough clarification to outstanding questions. Current evidence supports the recommendation of HDF as first line hemodialysis therapy, as seen in the United Kingdom^9,11^. In light of the findings of the CONVINCE trial, guidelines will likely be reviewed and HDF recommended globally.

HDF has been prescribed only recently in Brazil. Since its inclusion in the list of procedures of the National Supplementary Health Agency in 2021, the number of patients on HDF has grown exponentially among individuals with private health insurance. Data from the 2021 Census of the Brazilian Society of Nephrology revealed that about 8% of the patients with private health insurance were treated with HDF, while in the 2022 Census the number increased to 22%, making it more popular than peritoneal dialysis (7%) and home hemodialysis (0.3%)^12^.

The growth of HDF in Brazil requires the dissemination of information concerning the evidence supporting the therapy and the best clinical practices in use today. The implementation of HDF in Brazil was preceded by the organization of a multicenter randomized controlled trial (HDFit) comparing high-volume online HDF with high-flow HD without dialyzer reuse in both study arms, which offered local evidence for some comparisons^13^. This article provides technical information and evidence (including Brazilian data) to support the implementation, with quality and safety, of this new dialysis modality.

## Concept

HDF is a renal replacement therapy in which blood purification occurs concomitantly by diffusion and convection, the latter being responsible for a large part of the removal of toxins, especially those of greater molecular weight, such as β2-microglobulin (11,800 daltons)^1,2^.

Unlike conventional hemodialysis, the effectiveness and clinical benefits of HDF rely not only in diffusion, but also in convection, to remove a high volume of fluid^5–8,14,15^. The volume of fluid removed during an HDF session is generally greater than the overall volume of extracellular fluid; therefore, concomitant infusion of almost all of the fluid removed during treatment is required. The volume of fluid replaced is called substitution or infusion volume. At the end of an HDF session, a patient’s body weight is close to their dry weight. Online HDF was designed to provide proper volumes of substitution solution via an adequate, sterile, non-pyrogenic and low-cost dialysis fluid. HDF has become a viable option for the large-scale supply of maintenance dialysis^1,2,16–18^.

**Figure 1:**
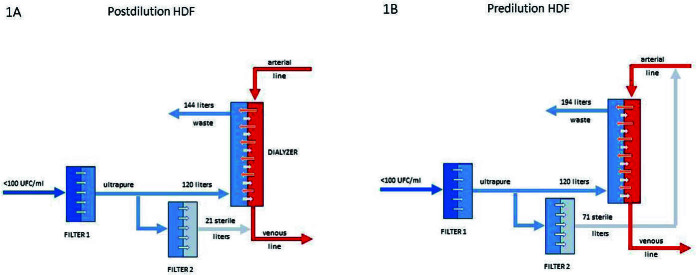
Simplified schematic design of post- (1A) and pre-dilution (1B) substitution fluid infusion modes.

In online HDF, part of the dialysis fluid undergoes double filtration to produce the substitution solution, which is infused into the patient. The remainder of the solution, which was not filtered in this second step, is used as an ultrapure dialysis solution to remove solutes by diffusion (Figure 1). When the substitution volume is infused into the dialysis line before the dialyzer, online HDF is called pre-dilution HDF; when it is infused after the dialyzer, it is called post-dilution HDF. Mixed-dilution HDF (part pre-dilution and part post-dilution) is not available in Brazil. Post-dilution HDF is the most commonly used method, since it optimizes the efficiency of solute removal and requires a smaller substitution volume. It is also the mode of substitution adopted in large controlled trials^1,9,16,18,19^.

Figure 1 shows a schematic design of how post- (Figure 1A) and pre-dilution (Figure 1B) online HDF work. In post-dilution, the patient undergoes an HDF session in which 24 liters are removed by convection, with 21 liters replaced. Thus, the negative balance is 3 liters, which is the interdialytic weight gain. The total volume of dialysis fluid used is 141 liters (120 as ultrapure dialysis solution, for solute clearance by diffusion, and 21 liters as sterile substitution solution). In pre-dilution infusion, 74 liters are removed by convection and 71 liters are replaced. Total use of dialysis solution is 191 liters (120 as ultrapure dialysis solution and 71 as sterile substitution solution).

## Water and Substitution Solution Quality

In Brazil, the water used in online HDF must meet the quality parameters set for conventional hemodialysis^2,18,20^. Resolution 11, dated March 13, 2014, establishes a limit of 100 CFU/mL for heterotrophic bacteria in microbiological analysis and of 0.25 EU/mL for endotoxins^21^. Under these conditions, the filters used in online HDF ensure the safety of therapy^1,2,16,18^.

The filters used in the production of sterile and pyrogenic ultrapure and substitution dialysis fluids are designed to meet the requirements set out in the ISO 23500-1:2019 and 23500-3:2019 standards. The guidelines stipulate that additional testing for endotoxins or bacteria is not required when validated systems and filters are used in online HDF. Cautionary measures include strict monitoring for water quality as described in Resolution 11, and the operation and monitoring of equipment and filters in accordance with manufacturer instructions.

## Equipment, Supplies and Dialyzers

Online HDF requires the use of appropriate dialysis equipment (machines), dialyzers and supplies^2,16,18^.

### Equipment

Online HDF-specific dialysis equipment plays an important role in treatment. A number of innovations have been introduced to control transmembrane pressure and infusion rates. HDF equipment available in Brazil include Fresenius 5008/5008S® (Fresenius), Dialog+HDF/Dialog IQ® (BBraun) and TR-8000® (Toray). Although they use different technologies, they can be used in pre- or post-dilution infusion and are equipped with automated ultrafiltration volume optimization systems and meet the safety requirements set for achieving high convection volumes^2,16,18^.

The 5008/5008S (Fresenius Medical Care, Germany) has an automatic substitution mode (AutoSub™), in which the substitution rate is automatically regulated in response to changes in several patient and treatment parameters throughout the session. These automated systems provide continuous adaptation of the substitution volume according to changes in blood viscosity within the dialyzer fibers, which are detected through the analysis of dynamic pressure pulse signals transmitted by the peristaltic movement of the pump. Thus, the convection volume to be achieved is not preset, and the equipment safely offers the highest convection volume possible for any given patient, depending on their hematological conditions, prescribed session duration, blood flow and dialyzer^2,16,18^.

The BBraun Dialog HDF® and Dialog IQ® (BBraun, Germany) continuously monitor the percentage of ultrafiltration in relation to the programmed substitution volume, allowing the optimization of the target convection volume. The Dialog HDF® controls the substitution volume based on the filtration fraction, which is calculated as the ratio between the ultrafiltration rate and blood flow. It automatically adjusts it according to variations in these parameters. The Dialog IQ® allows the infusion of pre- or post-dilution substitution volume, and is equipped with a biofeedback system (Biologic Fusion®) that continuously adjusts the ultrafiltration rate according to systolic blood pressure and relative blood volume, aiming to stabilize hemodynamic conditions and decrease the risk of hypotension during HDF^2,16,19^.

### Dialyzers and Lines

According to the EUropean DIALlysis group (EUDIAL), an organization established to encourage research, disseminate knowledge and contribute to education in the field of hemodialysis, the use of high-flow dialyzers is mandatory in HDF^2^. This type of filter has a water permeability coefficient (KUF) above 20 mL/h/mmHg per square meter of membrane surface area, a sieving coefficient for β2-microglobulin above 0.6, and enough selectivity to prevent excessive loss of macromolecules, such as albumin (68,000 daltons)^2^. Furthermore, dialyzers used in online HDF should preferably have fibers with larger lumen diameter (≥200 µm) than the ones used in conventional hemodialysis^2,16,18^. In HDF, most of the solutes are removed through convection. Removal of a large volume of fluid can cause hemoconcentration. Fibers with a larger diameter minimize this effect. Using conventional hemodialysis dialyzers with fibers of a smaller diameter increases the risk of clots and decreases the efficiency of online HDF^16–18^.

### Supplies

Larger gauge needles – 14G or 15G – are recommended to enable adequate convection volumes^16,18,22,23^. The dialysis solution formulations used in online HDF are the same as the ones used in conventional hemodialysis. Recommendations stress a preference for using solutions with glucose, potassium > 2 mEq/L and bicarbonate 32 mEq/L. Calcium concentration, usually set at 2.5 or 3 mEq/L, must be individualized^1,16,22,24^.

Table 1 shows an example of implementation of a high-volume online HDF program based on the prescription parameters used in the HDFit^25^ trial.

**Table 1:** HDF implementation protocol used by the dialysis centers included in the HDFit trial

Mode	Post-dilution high-volume online HDF
Anticoagulation	Heparin; the protocol in use in each dialysis center was implemented (initial bolus injection or continuous infusion pump) without dose changes
Needle	15G
Blood pressure (mmHg)	−200
Blood flow (mL/min)	400
Target convection volume (L)	22
Sodium (mEq/L)	138
Potassium (mEq/L)	2
Calcium (mEq/L)	3,0
Bicarbonate (mEq/L)	32
Glucose (mg/dL)	5.5

Source: Adapted from Guedes et al.^25^

Pharmacoeconomic studies comparing online HDF and conventional HD are scarce and complex to carry out, given that the cost composition of dialysis depends on the treatment protocol and local specificities, especially in relation to the reprocessing of supplies, water and hospitalization costs and prescribed drugs^26,27,28^. A study carried out by the National Institute for Health and Care Excellence (NICE) provided a robust analysis of HDF cost-effectiveness, which served as the basis for the development of public health system guidelines in the United Kingdom. For reasons tied to cost-effectiveness, online HDF has been recommended over HD in the United Kingdom^11^. This subject has not been the object of studies in Brazil. However, a cost-effectiveness analysis was included in the reports used to review the inclusion of online HDF in the list of procedures paid for by the National Supplementary Health Agency^29^. This analysis, with results similar to the ones found in the UK study, had as its main outcome years of life saved and included direct costs for renal replacement therapy (dialysis sessions), consultations and tests in accordance with the recommendations issued by the Ministry Health. The costs of adverse events, including death caused by stroke^29^, were also included. The impact on the supplementary health services budget was calculated over a five-year time horizon. The use of econometric techniques estimated an average annual population of 27,516 patients (individuals on dialysis with private health insurance in Brazil). As a result, an increase of R$24,189,417 in the first year and an accumulated increase of R$154,567,096 over five years were estimated, representing an average annual impact of R$30,913,419^29^. When incorporated into additional cost metrics related to the management of complications and years of life gained, the analysis indicated that HDF is cost-effective in the supplementary healthcare environment in Brazil. The additional pharmaco-economic analyses from the CONVICE and H4RT trials should provide more information to help define broader policies for access to HDF^9,10^.

## Appropriate HDF Dose

The appropriate high-volume online HDF dose was defined based on the results of the ESHOL trial^7^ and in the post hoc analyses of two other large clinical trials^5,6^. Confirmation was attained with the CONVINCE^9^ trial. HDF dose is calculated based on the desired convection volume, with a minimum value set around 23 liters per session for adult patients undergoing treatment three times a week. Patients on dialysis more than three times a week have an extrapolated target convection volume of 69 liters per week. Children and petite adults have a target weekly convection volume of 69 liters per 1.73 m^2^ of body surface area^4,15^.

## Strategies for Achieving Adequate Convection Volumes

Blood flow might be the single most important variable for achieving adequate convection volumes. There is a direct relationship between convection volume and effective blood flow^14,15,17,22^. To maintain an effective blood flow of at least 350 mL/min, good vascular access and monitoring arterial and venous pressure in the vascular access are required. Larger gauge needles are recommended for patients with arteriovenous fistulas or grafts^16,17,22,23,30^. In patients with a central venous catheter, achieving high blood flows depends on the model and diameter of the lumen of the catheter^30^. It is possible to achieve the desired convection volumes in patients equipped with these devices^9,25^.

Another important variable is the prescribed duration of online HDF sessions. They should last just as long as conventional HD sessions, i.e., around four hours for patients on dialysis three times a week. Shorter sessions or failure to comply with the prescribed session length hinders the achievement of the desired convection volume. Patients with hemoglobin levels above 12g/dL face additional obstacles to achieving the desired convection volume due to increased blood viscosity^31^.

**Figure 2:**
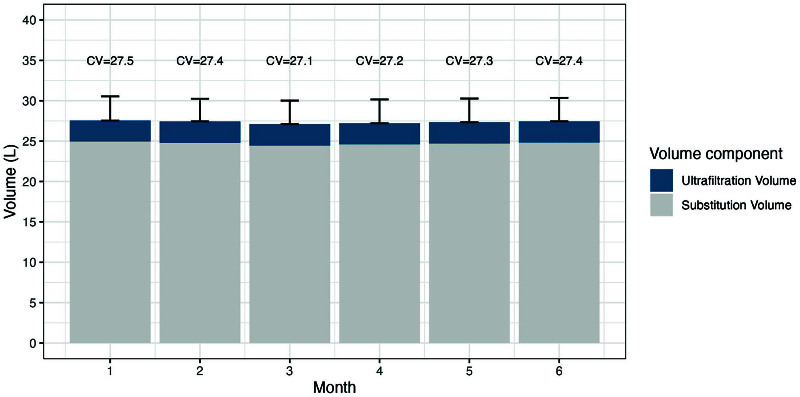
Percentage of patients who reached the pre-established convection volume in the HDFit trial (adapted from Guedes et al.^25^).

The HDFit^13^ trial enrolled 195 patients from 14 Brazilian dialysis centers randomized on a 1:1 ratio to online HDF or conventional high-flow hemodialysis, both with three 4-hour sessions per week. All patients had been on hemodialysis for at least three months and had vascular accesses with an effective flow >350 mL/min. The average convection volume achieved in patients receiving online HDF was 27.1 liters per session. Over the six months of the trial, approximately 99% of the patients allocated to the high-volume HDF group (n = 97) reached the pre-established minimum volume (Figure 2). This study demonstrated that, with an appropriate strategy, which included machines with an automated convection volume optimization function, dialyzers with greater internal volume, greater possible blood flow, adequate vascular accesses (including 7% of patients with long-term catheters), use of larger gauge needles and full adherence to treatment, the vast majority of the patients on hemodialysis were able to reach the minimum desired convection volume without difficulty. In this study, the treatment time needed to reach the target convection volume (Figure 2) was identical (around four hours per session) to the treatment time of patients in the high-flow HD group^25^.

When the minimum convection volume was not achieved, despite attempts to correct the items described previously, one option might be to increase the time on HDF, either by increasing the duration of sessions or the number of weekly sessions.

## Population Eligible for HDF

The National Supplementary Health Agency has not produced guidelines for online HDF. In principle, every patient on a regular conventional hemodialysis program is eligible for this new dialysis modality.

There is no consensus on whether a subpopulation or patients with certain clinical conditions might benefit most from high-volume HDF. Prior to the publication of the CONVINCE trial, due to possible financial limitations and budgetary impacts on supplementary healthcare in Brazil, limitations to HDF therapy have been suggested for specific groups of patients, including those diagnosed with cardiovascular disease; with evidence of complications due to retention of higher molecular weight toxins, such as β2-microglobulin; with kidney transplant restrictions; and growing children. These recommendations were based on evidence of limited quality from systematic reviews^32,33^, post hoc analyses of pooled data from different clinical trials^8,34^ and observational studies^35,36,37,38,39^. The CONVINCE trial, however, described a reduction in the risk of death in patients with clinical characteristics beyond the ones previously considered, suggesting that the benefits of HDF might reach a broader population^9^. In the near future, analyses of the pre-established secondary endpoints of this trial, which include patient-reported outcomes and pharmaco-economic analysis^40^, as well as data from the H4RT trial, which had broader patient inclusion criteria^10^, might produce evidence that favors the prescription of HDF to subgroups of patients with specific clinical or demographic characteristics. At the moment, the inability to achieve high convection volume is the only condition that limits the prescription of HDF therapy^5,6^.

## Impact of HDF on Outcomes

### Short-term Outcomes

Several studies have demonstrated that HDF allows better clearance of small and medium molecules when compared to high-flow hemodialysis^14,22,41,42 43,44^. In fact, greater removal of uremic toxins such as ß2-microglobulin, phosphorus, leptin, advanced glycation end products (AGEs) and inflammatory cytokines were observed in patients undergoing HDF^45,46,47,48,49,50^. These molecules have been associated with increased risk of cardiovascular events and death^51,52,53^.

The HDFit trial found significant reductions in serum phosphorus, indoxyl sulfate and p-cresyl sulfate levels. Increases in urea reduction rate (URR) and KTV, among others (Table 2), were also observed with online HDF^54^. Further analysis of the HDFit trial showed that online HDF changed the profiles of 16 metabolites in several pathways associated with the development of cardiovascular disease, when compared to HD^55^.

**Table 2:** Mean serum levels (before dialysis sessions) of uremic solutes in patients enrolled in the HDFit trial: HDF vs. HD

Biomarker	Impact of HDF compared to high-flow HD
Kt/V	Increase of 0.1 unit in 6 months
URR (%)	2.5% reduction in 6 months
Phosphorus (mg/dL)	Reduction of 0.4 mg/dL in 3 months
P-cresyl sulfate (umol/L)	Reduction of 2.4 umol/L per month
Indoxyl sulfate (umol/L)	Reduction of 2.9 umol/L per month
Beta-2 microglobulin (mg/L)	Reduction of 1.6 mg/L per month

Monthly averages describe the mean differences during six months of follow-up. URR: urea removal rate. *Subgroup of patients with median convection volume > 27.5 L/session.

Another element observed in the HDFit trial was the maintenance of hemoglobin levels with lower need of erythropoietin^25^. Similarly reported by other authors, the lesser need for erythropoietin is most likely due to the greater removal of inflammatory cytokines and uremic toxins with this dialysis modality, which consequently decreases erythropoietin resistance^56,57^.

### Outcomes Related to Patient Well-being

HDF can also improve patient-centered outcomes. Studies have shown that patients on HDF develop fewer signs and symptoms related to uremia and dialysis and score higher in quality-of-life tests^58,59^. The analysis of the primary endpoints considered in the HDFit trial indicated that online HDF led to increases in physical activity (based on the number of steps per 24 hours) at three months compared with high-flow HD. Although these results were not sustained at six months of follow-up, patients on HDF tended to report recovery times after dialysis 30 minutes shorter than their counterparts on high-flow HD^60^. The HDFit results, although not definitive, suggest that online HDF may improve the ability to perform activities of daily living – given the higher level of overall physical activity measured based on daily step counts – compared to standard therapy^60^.

Nevertheless, there is still doubt and discussion regarding the contribution of high-volume HDF in improving the quality of life of patients with kidney failure, as some trials have heterogeneous results^38,61^. Living with kidney failure can be extremely complex from a clinical, emotional and social point of view. The CONVINCE trial was designed to provide more comprehensive answers and explore the outcomes reported by patients through elaborate assessments, based on international initiatives that have recently provided better definitions for the most relevant domains and symptoms experienced by dialysis patients^62^.

### Long-term Outcomes

The long-term clinical benefits of HDF were initially derived from post hoc analyses of two large clinical trials: the Convective Transport Study (CONTRAST)^5^ and the Turkish trial Comparison of Post-Dilution Online Haemodiafiltration and Haemodialysis^6^. In the CONTRAST trial, patients treated with HDF who achieved the desired convection volume (> 22 liters) had a 39% reduction in the risk of death compared to patients treated with low-flow hemodialysis. Similarly, in the Turkish HDF Study, patients treated with HDF with convection volumes greater than 17 liters had a 46% reduction in the risk of death from all causes and a 71% decrease in cardiovascular deaths compared to patients treated with high-flow hemodialysis.

In the ESHOL^7^ trial, with a pre-defined goal to achieve a convection volume of 23 liters or more per session, a 30% reduction in the risk of death from all causes was found in the group treated with HDF compared to the patients treated with high-flow hemodialysis. A 55% decrease in the risk of infection-related death was also observed, along with a trend towards a reduction in cardiovascular death and a 22% reduction in the rate of hospitalizations from all causes.

Subsequently, the HDF Pooling Study^8^ meta-analysis, which included approximately 2,800 patients from three trials and a French study, reported a significant decrease in the risk of all-cause and cardiovascular deaths in patients prescribed HDF compared to patients receiving hemodialysis.

The CONVINCE trial randomized participants to treatment with high-flow hemodialysis or high-volume online HDF, aiming to achieve 23 liters/1.73 m^2^ of body surface area^62^. The primary endpoints were recently published, reporting a 23% reduction in the risk of death from all causes^9^. The absolute risk reduction for the primary endpoint of all-cause death was 4.6%, reflecting a number needed to treat (NNT) of approximately 22. Since the absolute risk difference is a function of the population’s baseline risk and given that the CONVINCE trial population had a relatively lower mortality rate than expected for a population of patients with renal failure on hemodialysis^9^, one might expect that the potential benefit in the real world, if the CONVINCE results are sustained, will be even greater in decreasing the absolute risk of death.

The secondary endpoints – which include death from specific causes, cardiovascular events, hospitalization for all causes and related to infection, patient-reported outcomes (PROMs) and cost-effectiveness of therapy – have not been published yet. The H4RT^10^ trial, scheduled to end in 2025, has as primary endpoint the combination of deaths not associated with cancer and hospitalization due to a cardiovascular event or infection. Secondary endpoints include death from any cause, mortality or cardiovascular disease or infection, health-related quality of life, treatment cost-effectiveness and environmental impact. These studies will be essential to clarify the points still under discussion and align the nephrology community regarding the benefits of high-volume online HDF for patients undergoing long-term dialysis treatment.

## Conclusion

Online HDF is a rapidly growing dialysis modality worldwide. The possible benefits of HDF in relation to conventional hemodialysis are based on the better efficiency of solute removal with the use of higher doses of convection combined with diffusion. The post-study analyses of the initial trials found that high convection volumes were associated with decreased mortality compared to conventional hemodialysis. The ESHOL and CONVINCE trials confirmed this benefit. Therefore, achieving a high convection volume should be a goal for every patient prescribed online HDF. The HDFit trial included multiple dialysis centers in Brazil and demonstrated that this goal can be achieved safely and without logistical barriers in the majority of the patients prescribed online HDF, bringing clear benefits through increased solute removal efficiency. Implementation of HDF using protocols based on the best evidence and clinical practices is essential to obtain the best clinical results from the point of view of efficacy and safety.
